# Synthesis and Bioactivity of Pyrazole Acyl Thiourea Derivatives 

**DOI:** 10.3390/molecules17055139

**Published:** 2012-05-03

**Authors:** Jian Wu, Qing Shi, Zhuo Chen, Ming He, Linhong Jin, Deyu Hu

**Affiliations:** State Key Laboratory Breeding Base of Green Pesticide and Agricultural Bioengineering, Key Laboratory of Green Pesticide and Agriculture Bioengineering, Ministry of Education, Guizhou University, Guiyang 550025, China; Email: jianwu2691@yahoo.com.cn (J.W.); shiqing982816@163.com (Q.S.); fcc.zchen@gzu.edu.cn (Z.C.); hmher@126.com (M.H.); fcc.jinlh@gzu.edu.cn (L.J.)

**Keywords:** pyrazole acyl thiourea derivatives, synthesis, antifungal activity, antiviral activity

## Abstract

Sixteen novel pyrazole acyl thiourea derivatives **6** were synthesized from monomethylhydrazine (phenylhydrazine) and ethyl acetoacetate. The key 5-chloro-3-methyl-1-substituted-1*H*-pyrazole-4-carbonyl chloride intermediates **4** were first generated in four steps through cyclization, formylation, oxidation and acylation. Thess were then reacted with ammonium thiocyanate in the presence of PEG-400 to afford 5-chloro-3-methyl-1-substituted-1*H*-pyrazole-4-carbonyl isothiocyanates **5**. Subsequent reaction with fluorinated aromatic amines resulted in the formation of the title compounds. The synthesized compound were unequivocally characterized by IR, ^1^H-NMR, ^13^C-NMR and elemental analysis and some of the synthesized compounds displayed good antifungal activities against *Gibberella zeae*, *Fusarium oxysporum*, *Cytospora mandshurica* and anti-TMV activity in preliminary antifungal activity tests.

## 1. Introduction

**Figure 1:**
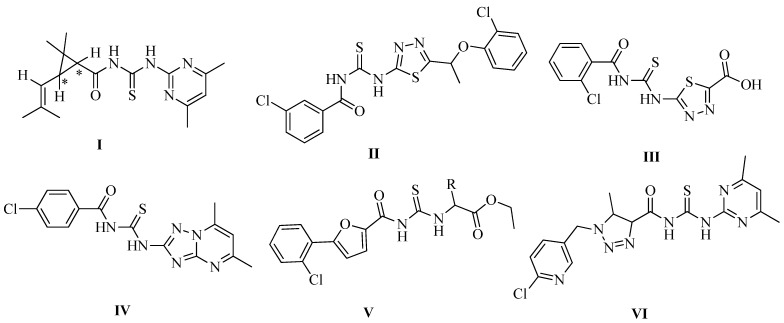
The structure of acyl thiourea derivatives **I**–**VI**.

Many acyl thiourea derivatives are well known to possess a wide range of bioactivities and are often employed as fungicidal, antiviral and regulating activities for plant protection in agriculture [1,2,3]. Some of them are employed as commercial insecticides [4], herbicides [5,6] and fungicides [7,8]. Several novel *N'*-(substituted pyrimidin-2-yl)-*N*-chrysanthemoylthiourea derivatives were synthesized by Xu’s group in 2005, and preliminary biological indicated that compounds **I** (Figure 1) exhibited 90% and 80% activity on *Bipolaris maydis* and *Powdery mildew* at 100 µg/mL, respectively [9]. Compounds **II** and **III** (Figure 1), arylthioureas containing 1,3,4-thiadiazole moieties, were found to show good plant growth regulator activity at 10 µg/mL [10,11]; Wang *et al.* [12] reported that acyl thiourea derivatives **IV** exhibited excellent selective herbicidal activity towards dicotyledons. In addition, some novel *N*-[5-(2-chlorophenyl)-2-furamidothiocarbonyl]-*L*-*α*-amino acid ethyl esters (**V**) (see Figure 1) were found to show good activity against K562 cells *in vitro* at 10 µg/mL [13]. Moreover, compound **VI** (Figure 1) that can be prepared by a multi-step reaction sequence using 2-chloro-5-(chloromethyl) pyridine, displayed 96.4% and 85.3% activities against rape root and barnyard root at 10 µg/mL, respectively [14]. 

**Scheme 1:**
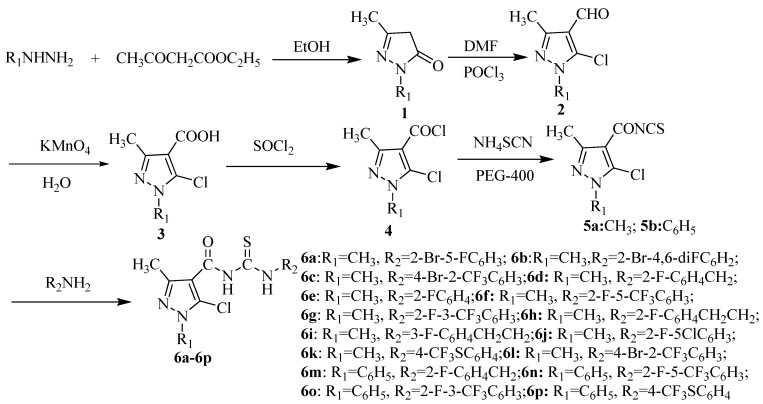
Synthetic route to compounds **6a**–**p**.

Pyrazole derivatives play an important role in the development of pesticides and medicines, which have become hot topics in recent years. Dozens of pyrazole products are available and widely used as fungicides, antiviral agents, analgesic agents, insecticides and herbicides [15,16,17]. In order to discover novel active compounds for use in agriculture, we sought to combine the active structures of thiourea and pyrazole to design and synthesize a class of novel pyrazole acyl thiourea derivatives. The synthetic route is shown in Scheme 1. All the compounds were unequivocally characterized by IR, NMR and elemental analysis. Preliminary bioassay tests indicated that most of the compounds exhibited antifungal activity against *G. zeae*, *F. oxysporum* and *C. mandshurica* and antiviral activity against TMV *in vivo* to a certain extent. Especially, compounds **6b**, **6h** exhibited slightly superior or similar activity on the corresponding fungi as compared to the commercial hymexazol.

## 2. Results and Discussion

### 2.1. Chemistry

The synthetic route to title compounds **6a**–**p** is depicted in Scheme 1. In present study, we have conducted our reaction using PEG-400 as solid-liquid phase transfer catalyst (PTC). PEG-400 as a phase transfer catalyst is indispensable for these reactions; it can easily react with NH_4_SCN to form a [PEG-400-NH4^+^] SCN^−^complex, which make it possible for SCN^−^ to readily react with 5-chloro-1,3-dimethyl-1*H-*pyrazole-4-carbonyl chloride and lead to the formation of intermediates **5a**. In the preparation of 5-chloro-1,3-dimethyl-1*H*-pyrazole-4-carbonyl isothiocyanate **5a**, phase transfer catalysis (PEG-400) and a conventional method were employed (see Table 1). From Table 1 we can see that the reaction in the presence of phase transfer catalysis (PEG-400) has many advantages such as low reaction temperatures and high yields. The use of PTC make the heterogeneous system react smoothly, the yield of the product be improved obviously [18,19]. The reaction of **5** with suitably substituted fluorinated primary aromatic amines afforded the desired acyl thioureas **6a**–**p** in good yields. 

**Table 1:** Phase transfer catalysis compared with conventional method for **6a**.

Method	Agent	Reaction Temperature	Reaction Time	Yield
phase transfer catalysis	NH_4_SCN/PEG-400	25 °C	3 h	82.6%
conventional method	NH_4_SCN	82 °C	3 h	51.2%

The structures of the compounds **6a**–**p** were established on the basis of their spectroscopic data. The IR (KBr) spectra of the target compounds **6a**–**p** showed characteristic N–H, C=O, and C=S absorptions at 3,200–3,430 cm^−1^, 1,660–1,690 cm^−1^ and 1,150 cm^−1^ respectively. The significantly reduced double bond character of the carbonyl group of the amide linkage accounts for the appearance of its stretching frequency at a lower wave number compared to a non-conjugated carbonyl. The ^1^H-NMR (CDCl_3_) spectra of these compounds exhibited the expected multiplet near *δ* 7.10–8.50 ppm due to the presence of aromatic protons. The two different N-H protons appeared as two broad singlets at *δ* 9.07–9.58 ppm and *δ* 10.66–12.81 ppm respectively, whereas the pyrazole methyl protons showed up near *δ* 2.50 ppm as a singlet. The characteristic chemical shifts for F/CF_3_ were also detected in the ^19^F-NMR (CDCl_3_) spectra of **6a**–**p**.

### 2.2. Antifungal Activity Bioassay

The *in vitro* antifungal screening data of the pyrazole acyl thiourea derivatives are provided in Table 2. It was observed that these synthesized compounds showed weak to good antifungal activities against the tested fungi at 100 μg/mL. Compounds **6b**, **6h** and **6l** were shown to inhibit the growth of *G. zeae* at 24.7%, 48.6%, and 22.9%, respectively; compounds **6b** and **6h** exhibited good activities on *F. oxysporum* at 50.3% and 57.9%, respectively, while compounds **6b** and **6h** inhibited the growth of *C. mandshurica* at 48.7% and 57.9%, respectively. These figures were slightly lower than those of hymexazol. Amongst the new products compound **6h** exhibited similar activities as hymexozole on the corresponding fungi. Although, a definite structure activity relationship could not be established with the limited experimental data and available compounds, it appears that with the incorporation of a 2-FC_6_H_4_CH_2_CH_2_- unit through R_2_ into thiourea in the resulting products **6** might have a positive influence, enhancing the antifungal activity of the designed compounds, From the activity differences between **6h** and **6i**, we can conclude that different fluorine atom (F) positions on benzene can result in different activity: when F was substituted at the 2-position of benzene (**6h**), it showed much higher activity than that of **6i** (where F was substituted at the 3-position of benzene), this may be caused by a steric effect on the internal hydrogen bond. 

**Table 2:** Inhibition effects of pyrazole acyl thiourea derivatives **6** on phytopathogenic fungi.

Compd. (100 µg/mL)	*G. zeae*	*F. oxysporum*	*C. mandshurica*
**6a**	2.84	1.46	−5.79
**6b**	24.7	50.3	48.7
**6c**	8.24	6.92	1.98
**6d**	12.8	−3.21	8.16
**6e**	9.57	−1.15	8.47
**6f**	−0.57	4.39	3.42
**6g**	17.6	−1.46	−3.68
**6h**	48.6	57.9	59.7
**6i**	19.9	4.09	6.57
**6j**	4.55	−3.80	−6.31
**6k**	15.4	11.8	7.91
**6l**	22.9	0.86	4.80
**6m**	−1.14	6.73	3.42
**6n**	5.85	−0.29	5.08
**6o**	7.18	5.19	11.9
**6p**	3.46	9.22	4.24
hymexazol	69.7	67.1	69.5

### 2.3. Antiviral Activity Bioassay

The antiviral activities of compounds **6a**–**p** against TMV were assayed by the reported method [20]. The results of the *in vivo* bioassay against TMV are given in Table 3. Ningnanmycin was used as a reference antiviral agent. The data provided in Table 3 indicate that the title compounds **6a**–**p** showed curative rates ranging from 19.76%–41.23%.

**Table 3:** The curative effect of the compounds **6a**–**p** against TMV *in vivo*.

Agent	Concentration (μg/mL)	Curative effect (%)	
**6a**	500	41.23	
**6b**	500	29.74	
**6c**	500	35.17	
**6d**	500	25.47	
**6e**	500	35.44	
**6f**	500	37.95	
**6g**	500	32.71	
**6h**	500	28.07	
**6i**	500	23.45	
**6j**	500	19.76	
**6k**	500	27.84	
**6o**	500	31.82	
**6m**	500	32.39	
**6n**	500	30.09	
**6o**	500	22.07	
**6p**	500	27.08	
Ningnanmycin	500	51.92	

## 3. Experimental

### 3.1. General

Unless otherwise stated, all the reagents and reactants were purchased from commercial suppliers. Melting points were uncorrected and were determined on a XT-4 binocular microscope (Beijing Tech Instrument Co., Beijing, China). The ^1^H-NMR and ^13^C-NMR spectra were recorded on a JEOL ECX 500 NMR spectrometer (Tokyo, Japan) at room temperature operating at 500 MHz for ^1^H-NMR, 125 MHz for ^13^C-NMR, and 470 MHz for ^19^F-NMR, using CDCl_3_ as solvents and TMS as an internal standard; infrared spectra were recorded in KBr on a Bruker VECTOR 22 spectrometer (Ettlingen, Germany); elemental analysis was performed on an Elemental Vario-III CHN analyzer (Hanau, Germany). The course of the reactions was monitored by TLC; analytical TLC was performed on silica gel GF_254_; column chromatographic purification was carried out using silica gel. The intermediates **1**–**3** were prepared according to the literature procedure [18].

### 3.2. Preparation of the Intermediate Acyl Isothiocyanates ***5***

The intermediates **3** (30 mmol) and thionyl chloride (15 mL) were placed in a dried round-bottomed flask containing a magnetic stirrer bar. The reaction mixture was stirred at 80 °C for 4 h until evolution of gas completely ceased. The excess thionyl chloride was carefully removed by rotary distillation to give a white solid to which were added ammonium thiocyanate (36 mmol) in acetonitrile (60 mL) and six drops of PEG-400. The solution was stirred at room temperature for 3 h, filtered and the solvent was removed on a rotary evaporator to afford a crude yellow solid which was further purified by column chromatography using a mixture of petroleum ether and ethyl acetate (v/v = 2:1) as developing solvent to give pure acyl isothiocyanate **5**. The appearance, melting points and yields of the intermediates **5a**, **5b** are provided in Table 4.

**Table 4:** Appearance, melting points and yields of intermediates **5a**–**b**.

Intermediate	R_1_	Appearance	m.p./°C	Yield/％
**5a**	CH_3_	Yellow solid	51~52 °C	82.6%
**5b**	C_6_H_5_	White solid	69~71 °C	81.7%

### 3.3. Preparation of Title Compounds ***6a**–**p***

To a well stirred solution of acyl isothiocyanate **5** (1.00 mmol) in THF (15 mL), a suitable fluorinated aromatic amine (1.20 mmol) was added. The mixture was stirred at room temperature for 4 h and monitored by TLC. After completion of the reaction, the solid was filtered; the solvent was evaporated and the crude product was purified by preparative TLC with a mixture of petroleum ether and ethyl acetate (v/v = 3/1–1/3) to give title compounds **6a**–**p**.

*1-(2-Bromo-5-fluorophenyl)-3-(5-chloro-1*,*3-dimethyl-1H-pyrazole-4-carbonyl)thiourea* (**6a**). White solid, yield 73.9%; m.p. 148–150 °C; ^1^H-NMR (CDCl_3_): *δ* 12.65 (1H, brs, CSNH), 9.28 (1H, brs, CONHCS), 8.33~6.88 (3H, m, Ph-H), 3.85 (3H, s, CH_3_), 2.50 (3H, s, CH_3_); ^13^C-NMR (CDCl_3_): *δ* 178.6, 160.9, 152.3, 137.7, 133.5, 127,6, 115.1, 114.9, 113.8, 113.6, 112.1, 108.7, 36.7, 14.8; ^19^F-NMR (CDCl_3_): *δ* −116.64; IR: *ν* 3396.6, 3145.9 (N–H), 1678.1 (C=O), 1159.2 (C=S) cm^−1^; Anal. Calc. for C_13_H_11_BrClFN_4_OS: C 36.85%, H 2.38%, N 13.22%. Found: C 37.00%, H 2.14%, N 13.38%.

*1-(2-Bromo-4*,*6-difluorophenyl)-3-(5-chloro-1*,*3-dimethyl-1H-pyrazole-4-carbonyl)thiourea* (**6b**). White solid, yield 74.1%; m.p. 84–86 °C; ^1^H-NMR (CDCl_3_): *δ* 11.74 (1H, brs, CSNH), 9.46 (1H, brs, CONHCS), 7.25~6.96 (2H, m, Ph-H), 3.84 (3H, s, CH_3_), 2.45 (3H, s, CH_3_); ^13^C-NMR (CDCl_3_): *δ* 181.3, 161.3, 152.1, 127.7, 124.0, 123.9, 122.8, 116.1, 115.9, 108.6, 104.7, 104.5, 104.3, 36.7, 14.7; ^19^F-NMR (CDCl_3_): *δ* −107.6, −109.1; IR: *ν* 3338.1, 3095.8 (N–H), 1668.4 (C=O), 1157.3 (C=S) cm^−1^; Anal. Calc. for C_13_H_10_BrClF_2_N_4_OS: C 39.30%, H 2.73%, N 13.81%. Found: C 39.30%, H 2.54%, N 13.98%.

*1-(4-Bromo-2-(trifluoromethyl)phenyl)-3-(5-chloro-1*,*3-dimethyl-1H-pyrazole-4-carbonyl)thiourea* (**6c**). White solid, yield 71.9%; m.p. 112–114 °C; ^1^H-NMR (CDCl_3_): *δ* 12.43 (1H, brs, CSNH), 9.36 (1H, brs, CONHCS), 7.89~7.73 (3H, m, Ph-H), 3.86 (3H, s, CH_3_), 2.49 (3H, s, CH_3_); ^13^C-NMR (CDCl_3_): *δ* 180.7, 161.3, 152.4, 135.3, 134.7, 131.4, 129.4, 127.6, 127.0, 126.8, 123.5, 121.3, 120.8, 108.7, 36.7, 14.8; ^19^F-NMR (CDCl_3_): *δ* −61.36; IR: *ν* 3373.5, 2993.5 (N–H), 1670.4 (C=O), 1126.4 (C=S) cm^−1^; Anal. Calc. for C_14_H_11_BrClF_3_N_4_OS: C 36.90%, H 2.43%, N 12.30%. Found: C 37.41%, H 2.10%, N 12.28%.

*1-(2-Fluorobenzyl)-3-(5-chloro-1*,*3-dimethyl-1H-pyrazole-4-carbonyl)thiourea* (**6d**). White solid, yield 82.2%; m.p. 121–122 °C; ^1^H-NMR (CDCl_3_): *δ* 10.92 (1H, brs, CSNH), 9.13 (1H, brs, CONHCS), 7.47~7.06 (4H, m, Ph-H), 4.96 (2H, d, *J* = 5.75 Hz CH_2_), 3.83 (3H, s, CH_3_), 2.44 (3H, s, CH_3_); ^13^C-NMR (CDCl_3_): *δ* 178.3, 161.6, 152.0, 130.5, 130.5, 129.9, 129.8, 127.6, 124.4, 124.4, 115.7, 115.5, 109.0, 43.5, 14.7; ^19^F-NMR (CDCl_3_): *δ* −117.88; IR: *ν* 3398.6, 3228.8 (N–H), 1668.4 (C=O), 1166.9 (C=S) cm^−1^; Anal. Calc. for C_14_H_14_ClFN_4_OS: C 49.34%, H 4.14%, N 16.44%. Found: C 49.57%, H 3.93%, N 16.93%.

*1-(2-Fluorophenyl)-3-(5-chloro-1*,*3-dimethyl-1H-pyrazole-4-carbonyl)thiourea* (**6e**). White solid, yield 74.5%; m.p. 152–154 °C; ^1^H-NMR (CDCl_3_): *δ* 12.56 (1H, brs, CSNH), 9.27 (1H, brs, CONHCS), 8.38~7.15 (4H, m, Ph-H), 3.87 (3H, s, CH_3_), 2.51 (3H, s, CH_3_); ^13^C-NMR: (CDCl_3_, 125 MHz): *δ* 178.8, 161.3, 152.3, 127.8, 127.7, 127.6, 126.0, 125.6, 124.0, 115.7, 115.6, 108.9, 36.7, 14.8; ^19^F-NMR (CDCl_3_, 500 MHZ): *δ* −124.43; IR (KBr): *ν* 3383.1, 2993.5 (N–H), 1666.5 (C=O), 1141.9 (C=S); Anal. Calcd for C_13_H_12_ClFN_4_OS: C 47.78%, H 3.70%, N 17.15%; Found: C 47.45%, H 3.83%, N 17.21%.

*1-(2-Fluoro-5-(trifluoromethyl)phenyl)-3-(5-chloro-1*,*3-dimethyl-1H-pyrazole-4-carbonyl)thiourea* (**6f**). White solid, yield 73.2%; m.p. 123–125 °C;^1^H-NMR (CDCl_3_): *δ* 12.80 (1H, brs, CSNH), 9.30 (1H, brs, CONHCS), 8.95~7.27 (3H, m, Ph-H), 3.85 (3H, s, CH_3_), 2.49 (3H, s, CH_3_); ^13^C-NMR: (CDCl_3_): *δ* 178.7, 161.4, 152.3, 127.7, 126.9, 126.8, 126.7, 126.4, 124.5, 124.3, 122.3, 116.1, 116.0, 108.6, 36.6, 14.7; ^19^F-NMR (CDCl_3_): *δ* −61.1, −126.5; IR (KBr): *ν* 3373.5, 3145.9 (N–H), 1670.3 (C=O), 1163.1 (C=S); Anal. Calcd for C_14_H_11_ClF_4_N_4_OS: C 42.59%, H 2.81%, N 14.19%; Found: C 42.76%, H 2.58%, N 14.34%.

*1-(2-Fluoro-3-(trifluoromethyl)phenyl)-3-(5-chloro-1*,*3-dimethyl-1H-pyrazole-4-carbonyl)thiourea* (**6g**). White solid, yield 74.9%; m.p. 139–141 °C;^1^H-NMR (CDCl_3_): *δ* 12.80 (1H, brs, CSNH), 9.31 (1H, brs, CONHCS), 8.75~7.26 (3H, m, Ph-H), 3.87 (3H, s, CH_3_), 2.51 (3H, s, CH_3_); ^13^C-NMR: (CDCl_3_): *δ* 180.1, 161.1, 151.8, 140.8, 140.8, 130.2, 130.1, 127.4, 124.4, 115.7, 115.6, 113.7, 113.5, 109.0, 36.7, 14.6; ^19^F-NMR (CDCl_3_): *δ* −62.0, −119.5; IR (KBr): *ν* 3387.0, 3145.9 (N–H), 1680.0 (C=O), 1143.8 (C=S); Anal. Calcd for C_13_H_11_BrClFN_4_OS: C 42.59%, H 2.81%, N 14.19%; Found: C 42.70%, H 2.61%, N 14.29%.

*1-(2-Fluorophenethyl)-3-(5-chloro-1*,*3-dimethyl-1H-pyrazole-4-carbonyl)thiourea* (**6h**). White solid, yield 79.3%; m.p. 97–99 °C; ^1^H-NMR (CDCl_3_): *δ* 10.66 (1H, brs, CSNH), 9.07 (1H, brs, CONHCS), 7.28~7.00 (4H, m, Ph-H), 3.92~3.89 (2H, q, CH_2_), 3.79 (3H, s, CH_3_), 3.06 (2H, t, *J* = 7.45 Hz, CH_2_), 2.40 (3H, s, CH_3_); ^13^C-NMR: (CDCl_3_): *δ* 180.0, 161.0, 151.6, 131.1, 128.5, 127.4, 125.2, 125.1, 124.3, 115.4, 115.3, 108.9, 45.5, 36.6, 27.8, 14.6; ^19^F-NMR (CDCl_3_): *δ* −118.20; IR (KBr): *ν* 3385.1, 3244.3 (N–H), 1666.5 (C=O), 1159.2 (C=S); Anal. Calcd for C_15_H_16_ClFN_4_OS: C 50.77%, H 4.55%, N 15.79%; Found: C 50.90%, H 4.16%, N 15.79%.

*1-(3-Fluorophenethyl)-3-(5-chloro-1*,*3-dimethyl-1H-pyrazole-4-carbonyl)thiourea* (**6i**). White solid, yield 78.8%; m.p. 117–119 °C; ^1^H-NMR (CDCl_3_): *δ* 10.68 (1H, brs, CSNH), 9.08 (1H, brs, CONHCS), 7.30~6.91 (4H, m, Ph-H), 3.93~3.89 (2H, q, CH_2_), 3.82 (3H, s, CH_3_), 3.03 (2H, t, *J* = 8.00 Hz, CH_2_), 2.43 (3H, s, CH_3_); ^13^C-NMR: (CDCl_3_): *δ* 180.1, 161.1, 151.8, 140.8, 130.2, 127.4, 124.4, 115.7, 115.6, 113.7, 113.5, 109.0, 46.6, 36.6, 34.1, 14.6; ^19^F-NMR (CDCl_3_): *δ* -112.97; IR (KBr): *ν* 3377.3, 3228.8 (N–H), 1672.3 (C=O), 1168.9 (C=S); Anal. Calcd for C_15_H_16_ClFN_4_OS: C 50.77%, H 4.55%, N 15.79%; Found: C 50.55%, H 4.21%, N 15.99%.

*1-(5-Chloro-2-fluorophenyl)-3-(5-chloro-1*,*3-dimethyl-1H-pyrazole-4-carbonyl)thiourea* (**6j**). White solid, yield 72.1%; m.p. 158–160 °C; ^1^H-NMR (CDCl_3_): *δ* 12.56 (1H, brs, CSNH), 9.27 (1H, brs, CONHCS), 8.38~7.15 (4H, m, Ph-H), 3.87 (3H, s, CH_3_), 2.51 (3H, s, CH_3_); ^13^C-NMR: (CDCl_3_): *δ* 179.0, 161.5, 152.3, 134.2, 127.7, 126.5, 124.2, 121.3, 121.1, 116.7, 116.6, 108.7, 36.7, 14.8; ^19^F-NMR (CDCl_3_): *δ* −116.72; IR (KBr): *ν* 3375.4, 2991.6 (N–H), 1670.4 (C=O), 1138.0 (C=S); Anal. Calcd for C_13_H_11_Cl_2_FN_4_OS: C 43.23%, H 3.07%, N 15.51%; Found: C 43.61%, H 2.96%, N 15.61%.

*1-(4-(Trifluoromethylthio)phenyl)-3-(5-chloro-1*,*3-dimethyl-1H-pyrazole-4-carbonyl)thiourea* (**6k**). White solid, yield 74.4%; m.p. 145–147 °C; ^1^H-NMR (CDCl_3_): *δ* 12.75 (1H, brs, CSNH), 9.21 (1H, brs, CONHCS), 7.92~7.52 (4H, m, Ph-H), 3.86 (3H, s, CH_3_), 2.49 (3H, s, CH_3_); ^13^C-NMR: (CDCl_3_): *δ* 178.2, 161.5, 152.3, 140.2, 137.0, 133.3, 127.7, 124.2, 121.6, 108.8, 36.7, 14.8; ^19^FNMR (CDCl_3_): *δ* −42.66; IR (KBr): *ν* 3410.2, 3032.1 (N–H), 1666.5 (C=O), 1139.9 (C=S); Anal. Calcd for C_14_H_12_ClF_3_N_4_OS_2_: C 41.13, H 2.96, N 13.70; Found: C 41.22, H 2.76, N 13.73.

*1-(4-Bromo-2-(trifluoromethyl)phenyl)-3-(5-chloro-3-methyl-1-phenyl-1H-pyrazole-4-carbonyl)* *thiourea* (**6l**): White solid, yield 75.9%; m.p. 142–144 °C; ^1^H-NMR (CDCl_3_): *δ* 12.43 (1H, brs, CSNH), 9.48 (1H, brs, CONHCS), 7.89~7.52 (8H, m, Ph-H), 2.60 (3H, s, CH_3_); ^13^C-NMR (CDCl_3_): *δ* 180.7, 161.5, 153.5, 136.9, 135.4, 134.7, 131.4, 129.7, 129.5, 127.4, 127.2, 126.9, 125.6, 120.9, 110.2, 15.0; ^19^F-NMR (CDCl_3_): *δ* −61.39; IR (KBr): *ν* 3373.5, 3119.2 (N–H), 1670.4 (C=O), 1149.6 (C=S); Anal. Calcd for C_19_H_13_BrClF_3_N_4_OS: C 44.08%, H 2.53%, N 10.82%; Found: C 44.13%, H 2.12%, N 10.72%.

*1-(2-Fluorobenzyl)-3-(5-chloro-3-methyl-1-phenyl-1H-pyrazole-4-carbonyl)thiourea* (**6m**). White solid, yield 82.9%; m.p. 106–108 °C; ^1^H-NMR (CDCl_3_): *δ* 10.93 (1H, brs, CSNH), 9.27 (1H, brs, CONHCS), 7.54~7.07 (9H, m, Ph-H), 4.97 (2H, d, *J* = 5.75Hz, CH_2_), 2.53 (3H, s, CH_3_); ^13^C-NMR (CDCl_3_): *δ* 180.1, 161.2, 152.9, 136.9, 130.5, 130.5, 129.9, 129.6, 129.4, 125.5, 124.4, 124.4, 115.7, 115.6, 110.6, 43.5, 14.8; ^19^F-NMR (CDCl_3_): *δ* −117.80; IR (KBr): *ν* 3394.7, 3248.1 (N–H), 1666.5 (C=O), 1165.0 (C=S); Anal. Calcd for C_19_H_16_ClFN_4_OS: C 56.64%, H 4.00%, N 13.91%; Found: C 57.07%, H 4.46%, N 14.22%.

*1-(2-Fluoro-5-(trifluoromethyl)phenyl)-3-(5-chloro-3-methyl-1-phenyl-1H-pyrazole-4-carbonyl)* *thiourea* (**6n**). White solid, yield 75.1%; m.p. 178–180 °C; ^1^H-NMR (CDCl_3_): *δ* 12.77 (1H, brs, CSNH), 9.35 (1H, brs, CONHCS), 7.92~7.52 (9H, m, Ph-H), 2.60 (3H, s, CH_3_); ^13^C-NMR (CDCl_3_): *δ* 178.7, 161.5, 153.4, 136.9, 129.7, 129.5, 127.4, 126.8, 126.7, 125.6, 124.5, 122.6, 116.2, 116.1, 110.2, 15.0; ^19^F-NMR (CDCl_3_): *δ* −61.98, −119.33; IR (KBr): *ν* 3215.3, 3076.5 (N–H), 1685.8 (C=O), 1157.3 (C=S); Anal. Calcd for C_19_H_13_ClF_4_N_4_OS: C 49.95%, H 2.87%, N 12.26%; Found: C 50.07%, H 2.73%, N 12.20%.

*1-(2-Fluoro-3-(trifluoromethyl)phenyl)-3-(5-chloro-3-methyl-1-phenyl-1H-pyrazole-4-carbonyl)* *thiourea* (**6o**). White solid, yield 76.2%; m.p. 146–148 °C; ^1^H-NMR (CDCl_3_): *δ* 12.81 (1H, brs, CSNH), 9.43 (1H, brs, CONHCS), 7.55~7.26 (8H, m, Ph-H), 2.61 (3H, s, CH_3_); ^13^C-NMR (CDCl_3_): *δ* 178.9, 161.5, 153.5, 136.9, 129.7, 129.5, 127.5, 125.5, 123.2, 117.5, 117.3, 110.2, 14.9; ^19^F-NMR (CDCl_3_): *δ* −61.09, −126.50; IR (KBr): *ν* 3221.7, 3086.2 (N–H), 1678.4 (C=O), 1153.2 (C=S); Anal. Calcd for C_19_H_13_ClF_4_N_4_OS: C 49.95%, H 2.87%, N 12.26%; Found: C 49.91%, H 2.77%, N 12.31%.

*1-(4-(Trifluoromethylthio)phenyl)-3-(5-chloro-3-methyl-1-phenyl-1H-pyrazole-4-carbonyl)thiourea* (**6p**). White solid, yield 75.7%; m.p. 109–111 °C; ^1^H-NMR (CDCl_3_): *δ* 12.77 (1H, brs, CSNH), 9.35 (1H, brs, CONHCS), 7.92~7.52 (9H, m, Ph-H), 2.60 (3H, s, CH_3_); ^13^C-NMR: (CDCl_3_): *δ* 178.1, 161.6, 153.2, 140.2, 137.0, 136.9, 129.7, 129.5, 127.4, 125.5, 124.1, 121.8, 110.2, 15.0; ^19^F-NMR (CDCl_3_): *δ* −42.64; IR (KBr): *ν* 3388.9, 3032.1 (N–H), 1674.2 (C=O), 1151.5 (C=S); Anal. Calcd for C_19_H_14_ClF_3_N_4_OS_2_: C 48.46%, H 3.00%, N 11.90%; Found: C 48.36%, H 2.97%, N 11.87%.

### 3.4. Antifungal Bioassays

The antifungal activity of all synthesized compounds was tested against *F. oxysporum*, *G. zeae*, and *C. mandshurica* by the poison plate technique [21]. All the compounds were dissolved in DMSO (10 mL) before mixing with Potato Dextrose Agar (PDA, 90 mL). The final concentration of the compounds in the medium was fixed at 100 μg/mL. The three kinds of fungi were incubated in PDA at 25 ± 1 °C for 5 days to get new mycelium for the antifungal assays, and then a mycelia disk of approximately 0.45 cm diameter cut from the culture medium was picked up with a sterilized inoculation needle and inoculated in the center of PDA plate. The inoculated plates were incubated at 25 ± 1 °C for 5 days. DMSO in sterilized distilled water served as control, while hymexazole was used as positive control for each treatment with three replicates being conducted for each experiment. The radial growth of the fungal colonies was measured on the sixth day and the data were statistically analyzed. The *in vitro* inhibiting effects of the test compounds on the fungi were calculated by the formula CV = (A − B)/A, where A represents the diameter of fungi growth on untreated PDA, B represents the diameter of fungi on treated PDA, and CV represents the rate of inhibition.

### 3.5. Antiviral Activity Bioassay

*Purification of tobacco mosaic virus*. Using Gooding’s method [22], the upper leaves of *Nicotiana tabacum L*. Inoculated with TMV were selected and ground in phosphate buffer, then filtered through double layer pledget. The filtrate was centrifuged at 10,000 g, treated twice with PEG and centrifuged again. The whole experiment was carried out 4 °C. Absorbance values were estimated at 260 nm using an ultraviolet spectrophotometer:



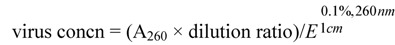



*Curative effect of compounds against TMV* in vivo. Growing leaves of *Nicotiana tabacum.* L. of the same ages were selected. The tobacco mosaic virus (concentration of 6 × 10^−3^ mg/mL) was dipped and inoculated on the whole leaves. Then the leaves were washed with water and dried. The compound solution was smeared on the left side and the solvent was on the right side for control. The local lesion numbers were then recorded 3–4 days after inoculation [23]. For each compound, three repetitions were conducted to ensure the reliability of the results, which were measured according to the following formula:



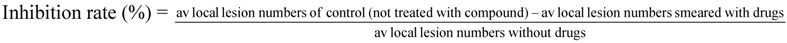



## 4. Conclusions

In the present study, a mild and effective method for the preparation of sixteen novel pyrazole acyl thiourea derivatives employing monomethylhydrazine (phenylhydrazine) and ethyl acetoacetate as the starting materials is described. The synthesized compounds were characterized by spectral data (^1^H-NMR, ^13^C-NMR, ^19^F-NMR, IR) and elemental analysis. The compounds were subjected to *in vitro* fungicidal activity assays against *G. zeae*, *F. oxysporum* and *C. mandshurica*. The results showed that the synthesized pyridazine compounds possessed weak to good antifungal activities against the tested fungi, with compounds **6b**, **6h** displaying good activity. Preliminary bioassays indicated that some of these compounds are also associated with good inhibitory activities against TMV at a concentration of 500 mg/L. Further studies are currently underway to establish a definite structure activity relationship. 
